# Pelvic pain in transmasculine adolescents receiving testosterone therapy

**DOI:** 10.1080/26895269.2022.2147118

**Published:** 2022-11-24

**Authors:** Dehlia Moussaoui, Charlotte V. Elder, Michele A. O’Connell, Ashleigh Mclean, Sonia R. Grover, Ken C. Pang

**Affiliations:** a Department of Paediatric and Adolescent Gynaecology, The Royal Children’s Hospital Melbourne, Parkville, Victoria, Australia; b Department of Paediatrics, University of Melbourne, Melbourne, Victoria, Australia; c Department of Endocrinology and Diabetes, The Royal Children’s Hospital Melbourne, Parkville, Victoria, Australia; d Murdoch Children’s Research Institute, Parkville, Victoria, Australia; e Department of Adolescent Medicine, The Royal Children’s Hospital Melbourne, Parkville, Victoria, Australia

**Keywords:** Adolescent, transgender, sexual and gender minorities, testosterone, pelvic pain

## Abstract

**Background:** Pelvic pain is a common complaint among individuals assigned female at birth. However, few studies have explored pelvic pain among transmasculine patients on gender-affirming testosterone treatment, and most of these were performed in adult populations. **Aims:** The aim of our study was to investigate the prevalence, risk factors, nature and treatment of pelvic pain among trans adolescents on testosterone. **Methods:** A retrospective cohort study was performed on all trans adolescents started on gender-affirming testosterone treatment at our institution between 2007 and 2020. **Results:** Among 158 trans adolescents who were started on testosterone therapy and followed-up for at least six months, 37 (23.4%) reported pelvic pain, with a median interval between testosterone initiation and reported onset of pain of 1.6 months (range 0.3-6.4). The prevalence of pelvic pain was higher in patients who were receiving menstrual suppression (n = 36, 26.3%) compared to those who were not (n = 1, 4.8%), giving a risk difference of 21.5% (95% CI 9.8% to 33.2%, p = 0.028). The most common descriptive terms were “cramps” (n = 17, 45.9%) and “similar to previous period pain” (n = 8, 21.6%). A range of different pharmacological strategies were employed, including paracetamol, NSAIDs, danazol, norethisterone, medroxyprogesterone, etonogestrel implant, intra-uterine device, goserelin and pelvic floor physiotherapy, with variable outcomes. **Conclusion:** In conclusion, we report here – in what is to our knowledge the first time – the prevalence rate of pelvic pain in trans adolescents on gender-affirming testosterone treatment, and observe that a quarter of them described pelvic pain. Limitations of our study include its retrospective nature, which is likely to be associated with under-reporting of pelvic pain, and the limited documentation of the nature and likely causes of this pain within the medical records. Prospective longitudinal studies to better understand the nature, etiology and optimal management of testosterone-associated pelvic pain are therefore warranted.

## Introduction

Transgender and gender diverse (hereafter, trans) individuals have a gender identity different from their sex assigned at birth. For trans individuals assigned female at birth, gender-affirming hormone therapy (GAHT) in the form of testosterone can induce physical changes (e.g. voice deepening, increased facial and body hair growth, change in body composition, and amenorrhea) that help to improve quality of life, mental health and gender dysphoria (Coleman et al., 2022; Hembree et al., 2017; van Leerdam et al., 2021). However, testosterone can also be associated with unwanted effects, such as acne, alopecia, vaginal mucosal thinning, erythrocytosis and hypertension (Hembree et al., 2017; Jarin et al., 2017; Olson-Kennedy et al., 2018; Stoffers et al., 2019).

Another unwanted effect that has been recently reported in trans individuals on testosterone is pelvic pain. Within the general adult population, pelvic pain has a prevalence of 25% among cisgender females and typically arises due to a diverse range of gynecological, musculoskeletal, gastrointestinal, neurological, infectious and urological causes (Ahangari, 2014; Lamvu et al., 2021; Latthe et al., 2006). Interestingly, higher rates have been reported in adult trans cohorts. For example, an online survey advertised on social media found a 69.4% rate of abdomino-pelvic pain after initiation of testosterone therapy among 183 transmen (Grimstad et al., 2020). In this study, pelvic pain was mostly described as “cramps” and related to sexual activity in half of participants. Furthermore, two recent retrospective cohort studies performed among adult trans men presenting for gender-affirming hysterectomy found that approximately half reported pelvic pain before surgery (Ferrando et al., 2021; Grimstad et al., 2019). Finally, the only published study that we are aware of to have examined pelvic pain in trans adolescents assessed dysmenorrhea in 35 transmasculine adolescents and young adults before the initiation of testosterone (Shim et al., 2020); twelve subsequently started testosterone, and pain persisted in four of them.

Given the limited nature of these previous studies, there are still many unanswered questions related to pelvic pain and testosterone therapy. Firstly, the design of these previous studies did not allow for a true prevalence rate to be accurately estimated given likely ascertainment biases. Secondly, the majority of these studies were focused on adults, and only one included adolescents (Shim et al., 2020). This is important since chronic pelvic pain has a major impact on health-related quality of life and school participation in adolescents (Gallagher et al., 2018; Nur Azurah et al., 2013). Moreover, the adolescent nervous system is thought to be more plastic and therefore potentially more susceptible to central sensitization from repeated episodes of pelvic pain (Vincent et al., 2011). Thirdly, the nature and likely etiology of pelvic pain in trans individuals on testosterone is poorly understood, and it remains unclear if any factors increase the risk of developing pelvic pain in this population. Finally, in the absence of a clear pathophysiology, evidence regarding treatment of pelvic pain in trans individuals on testosterone is sparse, and its outcome uncertain.

The aim of this study was therefore to investigate the prevalence, possible predictors, nature and treatment of pelvic pain among a cohort of trans adolescents on testosterone therapy at our institution.

## Methods

### Study setting

The Royal Children’s Hospital Gender Service (RCHGS) is a state-wide pediatric tertiary referral clinic for trans children and adolescents up to the age of 18 years, based in Melbourne, Australia.

### Study design

We performed a retrospective cohort study. Inclusion criteria were trans adolescents assigned female at birth, who commenced GAHT with testosterone at our institution between January 2007 and April 2020. Previous or current use of gonadotrophin releasing hormone agonists (GnRHa) did not affect study eligibility. Follow-up ended on 31 December 2021 or at the time of patient’s transition to adult-based services or loss to follow-up, whichever occurred first. Adolescents with a duration of follow-up less than 6 months after testosterone initiation were excluded to ensure that there was sufficient time to observe the onset of pelvic pain.

### Outcome measures

The primary outcome measure of interest was the presence of pelvic pain after the initiation of testosterone. To this end, medical records were manually reviewed and screened for mention of pelvic pain, abdomino-pelvic pain or pain in the lower part of the abdomen. Initiation of testosterone was defined as the date of the first script for the topical formulation or the date of the first injection for the intramuscular formulation.

Secondary outcome measures included description of the pain, the timing of its onset, treatment and outcome. Here, onset of pelvic pain was defined as the timing of onset reported by the patient, or as the date of the first documentation of pelvic pain in the medical chart if there was no mention of timing of onset.

Adolescents with documented pelvic pain were also compared to those without pelvic pain to determine whether particular factors were associated with a higher risk of pelvic pain. Specifically, demographic information (e.g. age) and data on use of GnRHa, menstrual suppression, testosterone formulation and dosage, and serum testosterone levels were also extracted from the medical records.

### Statistical analyses

Statistical analyses were performed using Stata 17.0 software (StataCorp LLC 2021, College Station, TX). Categorical data were reported as number and percentage, and continuous data as median and interquartile range (given the non-normal distribution). To compare different patient subgroups, risk difference (i.e. the difference between the risk of pelvic pain in two different groups) was calculated. The following subgroups were considered, based on information extracted from the medical charts: past use of GnRHa, use of hormonal medication to suppress menstruation (including levonorgestrel intrauterine device, etonogestrel implant, norethisterone, medroxyprogesterone acetate or oral combined contraceptive pill), formulation of testosterone at initiation (daily topical testosterone, 3-monthly intramuscular testosterone undecanoate, or 3-weekly intramuscular testosterone enanthate or mixed testosterone esters) and dose of testosterone at initiation (high versus low dose). ‘High’ dose of testosterone was defined as topical testosterone 32.5-50mg, IM testosterone enanthate or mixed testosterone esters 250 mg or IM testosterone undecanoate 1000 mg (doses equivalent to those used for standard maintenance dosing in adult men). ‘Low’ dose of testosterone was defined as topical testosterone 12.5-25mg, IM testosterone enanthate or mixed testosterone esters 125 mg or IM testosterone undecanoate 500 mg. Fisher’s exact test or chi-square test was used for the comparison of categorical parameters, and the Wilcoxon rank sum test was used for continuous parameters.

### Ethics

This study was approved by the Royal Children’s Hospital Human Research Ethics Committee (#36323).

## Results

Chart review of 158 transmasculine adolescents who met eligibility criteria was performed. The clinical characteristics of this cohort are provided in Table 1. Among the patients identified, 37 (23.4%) had documentation of pelvic pain after starting testosterone. Table 2 provides a comparison of those with and without pelvic pain based on various clinical features.

**Table 1.** Clinical characteristics of patient cohort.

	Total
Number of patients: n	158
Age at testosterone initiation: median (IQR) (years)	16.6 (1.4)
Past use of puberty blockers: n (%)	15 (9.5%)
Menstrual suppression: n (%)	137 (86.7%)
Menstrual suppression type: n (%)	
Levonorgestrel IUD	10 (7.3%)
Oral combined contraceptive pill	2 (1.5%)
Etonogestrel	3 (2.2%)
Norethisterone	106 (77.4%)
Medroxyprogesterone acetate (injection)	13 (9.5%)
Medroxyprogesterone acetate (oral)	3 (2.2%)
Type of testosterone at initiation: n (%)	
Topical	17 (10.8%)
3-weekly injection	63 (39.9%)
3-monthly injection	78 (49.4%)
Dose of testosterone at initiation: n (%)	
Low dose	74 (46.8%)
High dose	84 (53.2%)
Serum testosterone value: median (IQR) (nmol/l)	8.9 (7.5)
Follow-up after T initiation: median (IQR) (months)	22.1 (15.4)

Low dose = topical testosterone formulation 12.5–25mg, testosterone enanthate or mixed testosterone esters 125 mg, testosterone undecanoate 500 mg.

High dose = topical testosterone formulation 32.5–50mg, testosterone enanthate or mixed testosterone esters 250 mg, testosterone undecanoate 1000 mg.

**Table 2.** Comparison of adolescents with and without pelvic pain.

	No pelvic pain	Pelvic pain	Difference in risk of pelvic pain	P value
Number of patients: n (%)	121 (76.6%)	37 (23.4%)		
Age at testosterone initiation: median (IQR) (years)	16.8 (1.5)	16.3 (0.9)		0.08^d^
Follow-up after T initiation: median (IQR) (months)	19.3 (15.4)	26.4 (11.6)		0.004^d^
No past use of puberty blockers, n (%)	107 (74.8%)	36 (25.2%)	ref	0.196^b^
Past use of puberty blockers, n (%)	14 (93.3%)	1 (6.7%)	−18.5% (95%CI: −33.0 to −4.0%)	
No menstrual suppression, n (%)	20 (95.2%)	1 (4.8%)	ref	0.028^b^
Menstrual suppression, n (%)	101 (73.7%)	36 (26.3%)	21.5% (95%CI: 9.8% to 33.2%)	
Type of testosterone at initiation, n (%)				0.292^c^
3-monthly injection	63 (80.8%)	15 (19.2%)	ref	
3-weekly injection	47 (74.6%)	16 (25.4%)	6.2% (95% CI: −7.7% to 20%)	
Topical	11 (64.7%)	6 (35.3%)	16.1% (95% CI: −8.3% to 40.4%)	
Dose of testosterone at initiation, n (%)				
Low dose	54 (73.0%)	20 (27.0%)	ref	0.315^c^
High dose	67 (79.8%)	17 (20.2%)	−6.8% (95% CI: −20.1% to 6.5%)	

Low dose = topical testosterone formulation 12.5-25mg, testosterone enanthate or mixed testosterone esters 125 mg, testosterone undecanoate 500 mg.

High dose = topical testosterone formulation 32.5-50mg, testosterone enanthate or mixed testosterone esters 250 mg, testosterone undecanoate 1000 mg.

^a^ Statistical test: Wilcoxon rank sum test.

^b^ Statistical test: Fisher exact test.

^c^ Statistical test: Chi-square test.

^d^ Difference in medians.

The median age at testosterone initiation was 16.6 years old for the entire cohort (IQR 1.4, range 13.9-18.6), and was similar between those with pelvic pain and without pelvic pain.

The median follow-up after testosterone initiation was 26.4 months (IQR 11.6, range 8.3-45.5 months) in patients with pelvic pain and 19.3 months (IQR 15.4, range 6.1-57.4 months) in those without pelvic pain (p = 0.0067).

The most common form of testosterone at initiation was a 3-monthly injectable formulation (testosterone undecanoate; n = 78, 49.4%), followed by a 3-weekly injectable formulation (testosterone enanthate or mixed testosterone esters; n = 63, 39.9%) and then a topical testosterone formulation (n = 17, 10.8%). Pelvic pain was seen at roughly similar frequencies in those who started with topical formulations (n = 6, 35.3%), 3-weekly injections (n = 16, 25.4%), and 3-monthly injections (n = 15, 19.1%), with no evidence of a meaningful difference between groups (p = 0.292).

More than half of patients (n = 84, 53.2%) commenced ‘high’ dose testosterone, while the remainder (n = 74, 46.8%) were started on ‘low’ dose testosterone. Pelvic pain was noted at similar frequencies in those who started at ‘high’ dose (n = 17 of 84, 20.2%) or ‘low’ dose (n = 20 of 74, 27%), with no evidence of a meaningful difference between the two groups (p = 0.315).

A small number of patients within the cohort (n = 15, 9.5%) were on GnRHa at the time of testosterone initiation (median duration of 22.8 months, IQR 15.4, range 9.9-50.7). Only one of 15 (6.7%) of these patients reported pelvic pain, which was a lower rate than in those with no prior use of GnRHa (36 of 143, 25.2%), although the evidence supporting a meaningful difference between these two groups was poor (p = 0.196).

Most patients within the cohort (n = 137, 86.7%) were using menstrual suppression agents at testosterone initiation (Table 1). Patients who were receiving menstrual suppression displayed a higher rate of pelvic pain (n = 36 of 137, 26.3%) compared to those who were not on menstrual suppression (n = 1 of 21, 4.8%), giving a risk difference of 21.5% (95% CI 9.8% to 33.2%, p = 0.028).

The median interval between testosterone initiation and the onset of pain was 1.6 months (IQR 3.4, range 0.3-6.4). The pain intensity was quantified in 11 adolescents and, within this subset, it was reported as mild (1-3/10) in one and severe (7-10/10) in ten. The quality of pelvic pain experienced was described in multiple ways (Tables 3 and 4). The most common descriptive terms were “cramps” (n = 17, 45.9%) and “similar to previous period pain” (n = 8, 21.6%). Pelvic pain related to sexual activity was also reported in ten adolescents (27%), including directly after orgasm (or sexual dreams), during penetrative sex, as well as during arousal.

**Table 3.** Description and treatment of pelvic pain (n = 37 patients).

	Pelvic pain
**Pelvic pain description,** n (%)	
Cramps	17 (45.9%)
Similar to period pain	8 (21.6%)
Sex-related pain	10 (27%)
Post orgasm/after sexual dreams	*5 (13.5%)*
During penetrative sex	*3 (8.1%)*
Arousal	*2 (5.4%)*
Early morning/or waking up at night	5 (13.5%)
Suspicion of pelvic floor spasms	5 (13.5%)
Associated with breakthrough bleeding	11 (29.7%)
Associated with nausea and/vomiting	4 (10.8%)
Pain radiating into lower limbs	2 (5.4%)
**Treatment**, n (%)	
Paracetamol	9 (24.3%)
NSAID	9 (24.3%)
Codeine	2 (5.4%)
Danazol	4 (10.8%)
Progestogen (norethisterone, medroxyprogesterone)	10 (27%)
Levonorgestrel IUD	6 (16.2%)
GnRH agonist (zoladex, synarel)	3 (8.1%)
Amytriptilline	1 (2.7%)
Physiotherapy	1 (2.7%)
Heat pack	2 (5.4%)
Laparoscopy	1 (2.7%)

**Table 4.** Qualitative description of pelvic pain.

	Quote
Cramps	"Reminiscent of his previous period-related pain"
	"Period pain but without bleeding"
Sex-related pain	"In waves"
	"Sexual activity has become associated with deep sharp bilateral pelvic pain"
	"Triggers are arousal (not just secondary to penetration)"
	"New pelvic pain reported after orgasm"
	"Pain seems to occur after sexual dreams"
	"Attemps at penetrative vaginal sex with his partner have been associated with significant pain"
	"Unable to achieve penetration"
Temporal relationship with testosterone	"He describes some pelvic pain shortly after each injection"
	"Pain started within a few weeks of starting testosterone"
	"Since starting testosterone, pain appears to have become worse"

**Figure 1.**
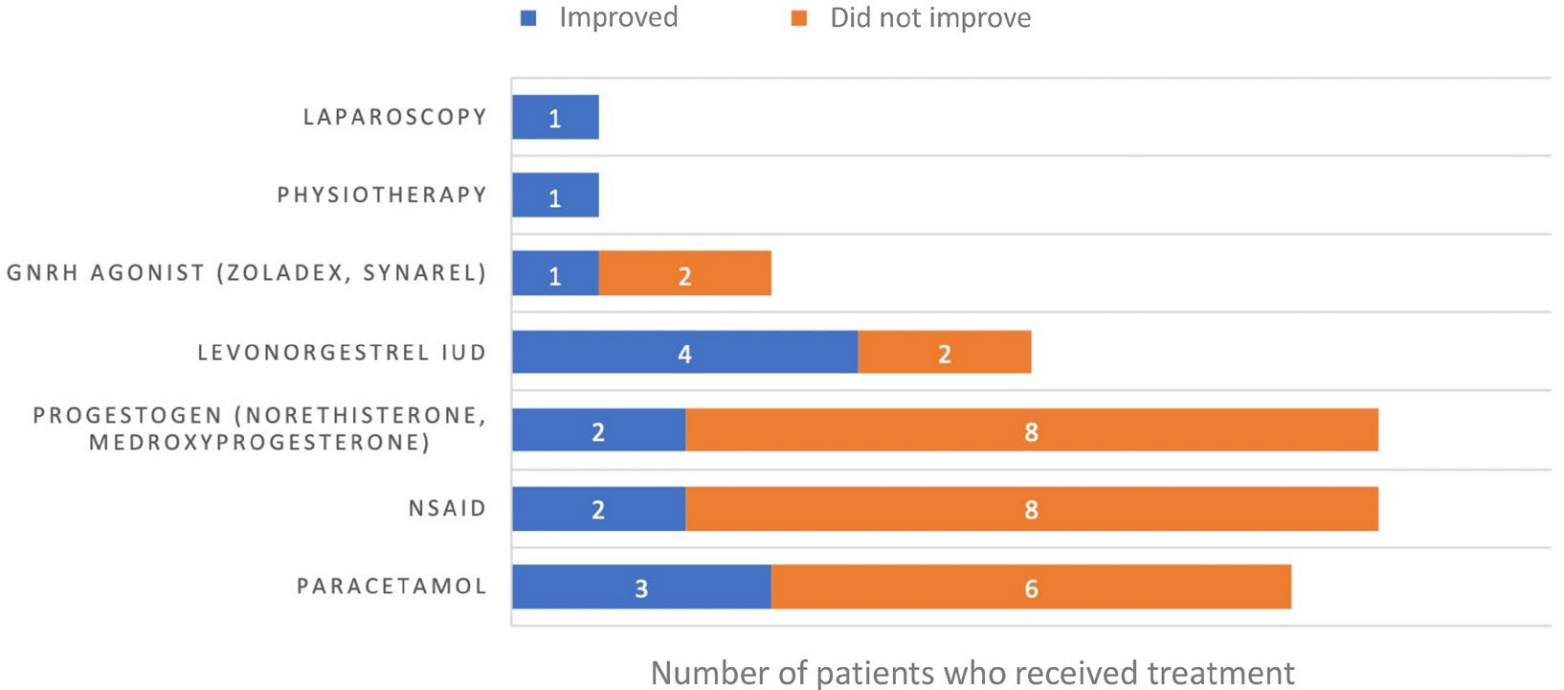
Outcome of pelvic pain with different treatment strategies. The number of patients who received different types of treatments for their pain is shown, and classified on the basis of whether improvement in pain was noted or not.

A range of different treatment strategies were employed to address the pain, most often initiated by the clinician (Figure 1). Nine patients (37.5%) were referred for adolescent gynecology consultation. The most frequent analgesics used were paracetamol and NSAID, which were both used in nine patients and provided documented relief in three and two patients respectively. Norethisterone and medroxyprogesterone acetate were the most common hormonal agents initiated in this setting to treat the pain, but only two out of ten patients reported an improvement. A levonorgestrel IUD was inserted in six patients, and pain improved in four of them. GnRHa were used in three patients, with pain improvement in one of them. A suspicion of pain related to pelvic floor spasms was mentioned for some patients (n = 5, 13.5%), and pelvic floor physiotherapy was suggested, but only one patient was documented to have attended these sessions with little reported improvement. One patient had a laparoscopic excision of endometriosis lesions, and pain was reported to be improved at five months. Overall, improvement or resolution of the pain was documented in eight patients (21.6%), after a median duration of 7.5 months (range 1-24).

## Discussion

In this retrospective cohort study of trans adolescents on testosterone therapy, we examined the prevalence and characteristics of pelvic pain.

We found that almost one in four transmasculine adolescents reported pelvic pain following testosterone commencement. The 23.4% prevalence of pelvic pain that we observed is to our knowledge the first such estimate in a cohort of trans adolescents. Moreover, although a few studies of transmasculine adults have explored the issue of pelvic pain associated with testosterone, none were designed to provide an accurate prevalence rate. For instance, Grimstad et al. conducted an exploratory survey in a community setting where participants were recruited through social media and snowball sampling to specifically recruit transmasculine individuals with abdomino-pelvic pain (Grimstad et al., 2020). They observed a 69.4% rate of abdomino-pelvic pain after testosterone initiation among 183 transmen (median age 40 years), which is higher than the rate we observed but is obviously subject to ascertainment bias and likely to be an overestimate of the true rate of pelvic pain. In a separate study, this same group assessed 94 trans men on testosterone (mean age 30 years) who were undergoing gender-affirming hysterectomy and observed a 57.7% rate of pelvic pain (Grimstad et al., 2019). Similarly, Ferrando et al. more recently assessed 67 trans men (mean age 29 years) undergoing gender-affirming hysterectomy (of whom 60 were on testosterone), and observed that half of the patients in their cohort reported pelvic pain preoperatively (Ferrando et al., 2021). However, the prevalence rates reported in these last two studies are again likely to be an over-estimate given the likely selection bias among patients presenting for hysterectomy, which is itself a treatment option for pelvic pain. The only study that we are aware of to have examined pelvic pain in trans adolescents explored dysmenorrhea in 35 transmasculine adolescents and young adults before the initiation of testosterone (Shim et al., 2020). Subsequently, twelve started testosterone and pelvic pain persisted in four (i.e., one third). This proportion is slightly higher than the one observed, but making a direct comparison is problematic since this sub-cohort on testosterone was small and included only those with existing dysmenorrhea.

In our study, we were also able to gain some understanding of the characteristics of pelvic pain in trans adolescents on gender-affirming testosterone therapy. Within our cohort, pelvic pain occurred soon following testosterone initiation with a median interval of 1.6 months. In comparison, Grimstad et al. reported a longer median interval of one year between testosterone initiation and pelvic pain onset (Grimstad et al., 2020), but their study relied on retrospective self-recall from their respondents and may thus be subject to greater bias. Interestingly, sexual activity was reported as a pain trigger in 27% of our patients. Although sexual activity was discussed alone with the adolescent in a confidential environment, there is the potential that patients may have not disclosed this (e.g. due to embarrassment) and the true rate might be higher. Consistent with this, Grimstad et al. observed that half of their respondents reported sexual intercourse as a precipitating factor for pelvic pain (Grimstad et al., 2020). Within our cohort, the most common description of pelvic pain was “cramps”, which is consistent with previous literature (Grimstad et al., 2019, 2020). When considering the intensity of pelvic pain, we observed that the majority of patients for whom we had suitable data rated their pain as severe. However, these results should be regarded with caution since it is possible that patients with more severe pain were more likely to have this intensity documented by their clinicians. Another limitation in this regard is that we were unable to adequately assess the broader impact pelvic pain (e.g. on relationships, school participation and quality of life), since this was not consistently explored and/or documented in the medical record. Evidence from studies on pelvic pain in cisgender female adolescents indicates a negative impact on health-related quality of life (Gallagher et al., 2018; Nur Azurah et al., 2013) and it would be important to assess this in future studies.

One of the aims of our study was to look at possible predictors of pelvic pain in trans individuals on testosterone. In this regard, it was notable that the risk of pelvic pain was >5x higher in individuals who were prescribed additional agents for menstrual suppression (26.3%) compared to those who were not (4.8%, p-value = 0.028). One possible explanation for this is that patients with a history of dysmenorrhea may have been more likely to have received menstrual suppression, in which case this group may have been more susceptible to pelvic pain in the first place. Unfortunately, past history of dysmenorrhea was insufficiently documented in most patients’ records, so we were unable to examine this as a potential independent risk factor. Within our cohort, there was also insufficient evidence to support any differences in the prevalence of pelvic pain and prior use of GnRHa, starting testosterone dose or type of testosterone formulation. Nevertheless, our observation that the proportion of pelvic pain was >3 times higher in patients who had not received GnRHa (compared to those who had) was interesting. At our institution, GnRHa are typically only administered to trans individuals in the early-mid stages of puberty who have yet to reach menarche. In this context, pubertal suppression and the resultant lack of a subsequent menarche might lower the risk of central sensitization related to dysmenorrhea and could act as a protective factor for the development of subsequent pelvic pain. Regardless, future studies to compare the impact of prior GnRHa use on pelvic pain in trans individuals on testosterone are warranted, and would ideally include greater numbers of patients on GnRHa, since the small numbers of this subgroup in our cohort were a key limitation.

Finally, our study demonstrated use of a wide range of treatments for pelvic pain in trans adolescents on testosterone. However, given our study design, we were unable to comment on the relative efficacy of any of these treatments. The lack of standardized treatment approaches that we observed in our cohort has also been reported by others (Grimstad et al., 2020; Shim et al., 2020), and presumably stems from our poor understanding of why pelvic pain arises in these patients. In this regard, the retrospective nature of our study meant that we were unable to establish the likely causes of pelvic pain within our cohort, but different possibilities were mentioned in certain cases. For instance, pelvic floor muscle dysfunction was suspected as a cause in some of our patients. This possibility has been suggested by others (Ferrando et al., 2021; Grimstad et al., 2020; Shim et al., 2020), with the proposed mechanism relating to a testosterone-induced increase in muscle mass modifying the postural carriage, but definitive evidence is lacking. Another possible origin of the pelvic pain that we observed is the uterus and this has also been mentioned by some authors (Ferrando et al., 2021; Grimstad et al., 2020). Histopathological studies have found inconsistent effects of testosterone on the endometrium, with some patients displaying an atrophic endometrium and others a proliferative endometrium (Grimstad et al., 2019; Hawkins et al., 2021). The expected increase in prostaglandin signaling associated with the latter might be expected to help drive pelvic pain (Dawood, 2006; Evans & Salamonsen, 2012; Wu et al., 2010), but further studies would be required to test this hypothesis. Related to this, endometriosis has also been proposed as a potential cause of pelvic pain in transmen on testosterone, but again empirical evidence is lacking. For instance, although Ferrando et al. found histologically confirmation of endometriosis in one third of their patients at the time of their hysterectomies, there was no statistically significant association between the presence of endometriosis lesions and pelvic pain (Ferrando et al., 2021). Similarly, Shim et al diagnosed endometriosis at laparoscopy in one transmasculine young people after commencement of GAHT, but these authors also noted that testosterone might be expected to have efficacy in ameliorating endometriosis-associated symptoms similar to danazol (Shim et al., 2020).

Apart from the limitations already mentioned, there are several other limitations to our study that are worth mentioning. Firstly, given its retrospective nature, there is a strong possibility that pelvic pain in this cohort was under-reported, so the true prevalence of pelvic pain in trans adolescents on testosterone therapy might be higher than the 23.4% we observed. Such under-reporting may be due to not only a failure of patients to mention such pain themselves but also a lack of exploration and/or documentation by the medical team. In this regard, improving awareness of the possible link between testosterone and pelvic pain will be important for patients and clinicians alike not only to obtain more accurate data but also to assist with management of this pain as part of routine follow-up care. Secondly, descriptions of the pelvic pain—including its nature, severity, associated features and responses to treatment—were often poorly documented in the medical records of our patients and thus limit the scope and generalizability of our findings in this area, while also making it difficult to make inferences about likely etiology. Similarly, the retrospective nature of this study and the inconsistent documentation of preexisting pain did not allow us to differentiate between new onset pelvic pain and persisting pelvic pain that was already present before testosterone initiation. Thirdly, we observed that adolescents with pelvic pain following testosterone initiation had significantly longer follow-up than adolescents without such pain. This might reflect the possibility that longer follow-up allows greater time to elicit reports of such pain, in which case one would expect to potentially uncover more instances of pelvic pain with future follow-up. However, it’s also plausible that adolescents without pelvic pain were more quickly transferred to the care of their general practitioner and other adult-based services, while adolescents with pelvic pain were more likely to be retained for care within our institution to help manage this issue. Nonetheless, we are aware that the median follow-up of our cohort was only 22.1 months (which was largely a function of the fact that the median age at testosterone initiation was 16.6 years and our service generally sees adolescents up to the age of 18 years before transfer to adult services). This duration could rightly be considered short in terms of the onset and development of pelvic pain, which is generally considered a chronic condition. Looking ahead, prospective longitudinal studies over a longer period of time will therefore be important to better understand the development, evolution and outcomes of pelvic pain in this population.

## Conclusion

Our data suggest that pelvic pain is a relatively common occurrence in trans adolescents prescribed testosterone, and we therefore recommend that the possibility of pelvic pain be routinely explored in this clinical setting. Looking ahead, we also recommend prospective longitudinal studies to better understand the potential association between testosterone and pelvic pain, including its risk factors, common causes and optimal management. In time, this will hopefully enable development of a standardized approach to the prevention and management of pelvic pain in trans individuals receiving testosterone.
